# Research on the influence of virtual reality on muscle fatigue during rowing ergometer exercise – pilot study

**DOI:** 10.1371/journal.pone.0342166

**Published:** 2026-02-06

**Authors:** Natalia Daniel, Jerzy Małachowski, Kamil Sybilski, Dariusz Siemiaszko, Kamil Klicki

**Affiliations:** 1 Military University of Technology, Faculty of Mechatronics, Armament and Aviation, Institute of Rocket Technology and Mechatronics, Warsaw, Poland; 2 Military University of Technology, Faculty of Mechanical Engineering, Institute of Mechanics & Computational Engineering, Warsaw, Poland; 3 Military University of Technology, Faculty of Chemistry and New Technology, Department of Functional Materials and Hydrogen Technology, Warsaw, Poland; Roma Tre University: Universita degli Studi Roma Tre, ITALY

## Abstract

The growing popularity of virtual reality (VR) applications has been reflected in numerous studies, particularly those examining the effects of VR on the human body, physical activity, and sports training. Comparative research suggests that simulated environments can influence physiological and psychological responses in distinct ways. The integration of VR with electromyographic (EMG) systems opens new opportunities to study biofeedback and muscle activation during exercise in real-time. However, only a limited number of studies have provided quantitative data on muscle fatigue. In the present research eight healthy male participants from previously described studies were examined using a VR environment to explore muscle fatigue. EMG signals were recorded from three muscle groups, and knee flexion angles were monitored. A VR simulation developed in Unreal Engine 5 was designed to reproduce a natural river scene for rowing training. The Discrete Wavelet Transform (DWT) was applied to both previously collected and VR-based data, calculating median frequency (MDF) distributions and linear regression for lower extremity muscles. Wilcoxon signed-rank tests comparing VR and non-VR conditions for the measured muscles: the Rectus Femoris, Biceps Femoris, and Gastrocnemius Lateralis, did not reveal statistically significant differences (all p > 0.05). Although no significant differences were observed, the proposed methodology introduces a valuable framework for quantitative fatigue assessment. By integrating VR with EMG analysis, this approach provides new perspectives for investigating muscle fatigue and its modulation in immersive environments.

## Introduction

The influence of virtual reality (VR) simulated environments on the human physiological and biomechanical response has been widely investigated in numerous scientific studies, particularly those addressing applications in sports science and physical activity [1–3]. The use of VR technology in the context of fitness training [4] and physical sports activities [5,6] is becoming more popular. Furthermore, the scientific literature provides a comprehensive description of the impact of simulated environments on sports experiences, including studies focused on the use of rowing ergometers [7,8]. In the context of VR, the rowing ergometer serves as an effort interface that can be transformed into a virtual boat, allowing users to navigate through a designed environment. This configuration offers a practical tool for monitoring various physiological and biomechanical indicators during exercise, which are important for analysing training efficiency and optimising exercise techniques [9].

The use of VR environments has the potential to induce a spectrum of adverse effects affecting users [10–12]. One of the main adverse effects associated with VR environment exposure is cybersickness [13], characterised by symptoms such as increased fatigue, disorientation, reduced motivation, and other undesirable outcomes [14]. Comparative studies have also examined the differential impact of various VR settings on physiological and psychological responses [15,16]. For instance, a recent study compared simulated forest (natural) environments and urban environments to assess their effects on participants’ physiological and psychological responses. The study’s findings indicated that, with respect to physiological responses such as systolic blood pressure and heart rate, both environments produced a general decrease over time. However, with regards to psychological responses, simulated urban settings were associated with increased fatigue and decreased self-esteem, whereas simulated forest environments enhance vigour and reduced negative emotions, such as disorientation and fatigue [17].

An interesting aspect of VR usage is its integration with other systems. For example, VR systems used in conjunction with electromyographic (EMG) systems for biofeedback studies have been applied in various fields, including rehabilitation [18,19], prosthetics [20] and sports [21]. EMG allows researchers to monitor changes in muscle activity during experiments and helps identify specific muscle movements [22–24]. Studies combining VR and EMG systems often focus on observing changes in emotions, cognitive functions, and physiological reactions in response to virtual environments [25]. In studies examining muscle activity using EMG during VR scenarios with various types of games, researchers observed the impact of dynamic scene elements as potential stimuli on strength training [26]. Furthermore, within VR–EMG studies, systems have been developed to quantitatively estimate muscle effort, such as when lifting weights of different masses at a specified joint angle [27]. Another research direction involving e EMG analysis in VR compared non-immersive 2D environments with an immersive VR condition to assess the participants’ performance during cycling. In this study, the muscle activity of the vastus lateralis and gastrocnemius muscles was measured using surface electromyography. As a result, both environments were found to affect participants’ performance, although prolonged exposure to VR could also lead to reduced exercise participation [28].

The integration of VR with EMG measurement opens up new research directions. The virtual environment itself can generate a range of effects, both beneficial and adverse, in relation to participant fatigue. However, only a limited number of studies have provided a quantitative analysis of muscle fatigue using EMG data in a VR context. The calculations in the current study are based on previous research [29,30], in which muscle fatigue was quantitatively assessed using a computational algorithm employing the discrete wavelet transform (DWT). This study followed a similar experimental design, measuring muscle fatigue in eight athletes from a university sports club across three muscle groups: Rectus Femoris (RF), Biceps Femoris (BF), and Gastrocnemius Lateralis (GAS). A custom-designed VR environment depicting a natural landscape was used, where the rowing ergometer simulated a boat, allowing the participants to row through a virtual scene filled with mountains and vegetation. This experimental setup aimed to determine whether the level of muscle fatigue differed when training occurred in a more immersive, naturalistic VR environment. In contrast to earlier studies that primarily focused on static VR scenes or short-term trials, the present research adopts a dynamic approach simulating extended rowing activity within a natural setting. Moreover, the application of advanced EMG data analysis technique, such as the discrete wavelet transform (DWT), enables a more detailed and accurate assessment of muscle fatigue progression. This study also distinguishes itself by focusing on endurance-oriented rowing exercises, an area rarely explored in the context of VR and EMG research. It provides valuable insights into physiological adaptations and muscle fatigue dynamics. As highlighted in the literature, investigations into the specific influence of VR on muscle fatigue require longer experimental durations to capture realistic muscle response patterns [31]. To address this, the present work compares quantitative results obtained in a traditional, non-VR environment [29] with those measured in the newly developed VR-based setting. Overall, the study offers an innovative perspective on muscle fatigue and endurance during ergometer rowing providing comparative evidence on muscular response and performance efficiency in VR versus non-VR conditions.

## Materials and methods

The experimental procedures applied in this study were adapted from the previously reported work [29] and further integrated into a VR environment. The participants were recruited according to the inclusion criteria defined in the earlier study and were identical to those who took part in the previous experiment [29]. The main objective of the present research was to evaluate and examine the effect of the VR environment on muscle fatigue within this selected group of participants.

### Participants

The study involved eight healthy young male volunteers, all the members of the Rowing Ergometer Section at the Military University of Technology (pl. AZS WAT). As students of the military university, the participants shared similar daily routines, disciplined training regimes, and membership in the same specialised sports section, which ensured a high degree of homogeneity within the study group. Key inclusion criteria were male sex, 20–25 years old, active membership in the university rowing section, and general good health. The recruitment period for this study began on July 4, 2022, and ended on December 15, 2022. None of the participants reported a history of musculoskeletal disorders. The main characteristics of the group were as follows (mean ± standard deviation): age 21.5 ± 1 year; height 183 ± 6 cm; body weight 86.5 ± 8 kg; and body mass index (BMI) 25.8 ± 1.89. Additionally, each participant maintained a high level of physical activity throughout the week, consistent with their intensive training routine of the university’s sports section. This demanding activity level, together with dietary patterns shaped by the structured and disciplined lifestyle typical of military students – whose diet was determined to have a neutral impact on health, further emphasised the homogeneity of the study group in terms of physical conditioning and overall health. Exclusion criteria included a history of cardiovascular disease, lower limb pathologies, previous lower limb surgeries, or neurological disorders. Participant eligibility regarding these criteria was verified by a sports medicine physician. The data collection for the VR condition was conducted following a time interval after the completion of data collection for the non-VR condition. The period between the end of the first and the start of the second experimental phase was approximately six months. To minimise the potential impact of this time interval on the comparability of results and to address potential confounding factors related to the passage of time, the study participants were continuously monitored during this period, under the supervision of the rowing section coach. This monitoring included regular verification of dietary adherence, confirmation of body weight stability, and assessment of maintaining a constant level of physical performance. The latter was evidenced by participants achieving similar times during standardised rowing trials. The key performance indicators recorded by the rowing ergometer remained stable throughout the period separating the two experimental phases.

The participants were fully informed about the measurement procedure and the potential risks associated with the testing. Each participant confirmed their voluntary participation by signing a written consent. The study was conducted in accordance with the decision of the Research Ethics Committee for Human Studies at the Warsaw University of Life Sciences (pl. SGGW), approval number 19/22.

### Methods

**Fig 1:**
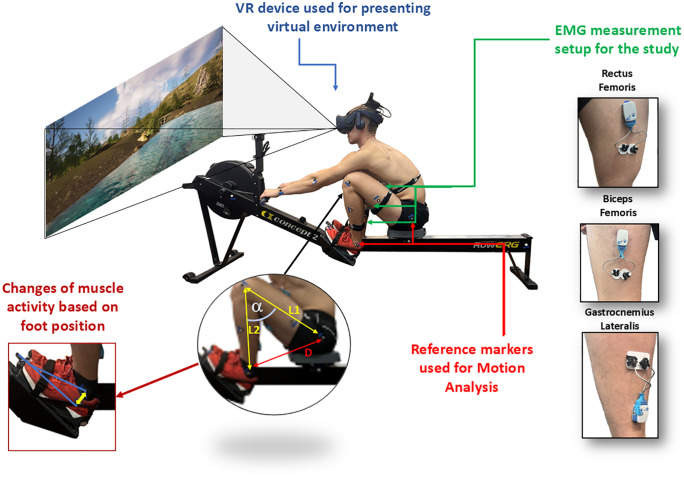
The scheme illustrates the applied experimental procedure.

In this study, eight participants from the AZS WAT sports section performed rowing exercises on a rowing ergometer within a VR environment. The method employed in this study is illustrated in Fig 1.

The virtual environment was displayed using HTC VIVE Pro 2 headsets. Skin preparation was carried out in accordance with SENIAM standards – prior to electrode attachment, the skin was shaved and thoroughly cleaned with an alcohol-ether solution to reduce impedance. Electrode placement, equipment configuration, participant positioning, foot adjustments for optimal muscle activation and signal filtering were all performed according to the same standardised procedures. Each EMG signal was pre-processed via a Butterworth band-pass filter (20–500 Hz) to remove low- and high- frequency artifacts while preserving the physiological frequency content. During the rowing exercises, EMG signals were recorded from three muscle groups: Gastrocnemius Lateralis (GAS), Biceps Femoris (BF) and Rectus Femoris (RF). The electrodes, positioned 20.0 mm apart (centre to centre), were placed along the muscle bellies identified by palpation in both legs [32]. EMG data were collected using a 32-channel Ultium EMG system (Noraxon, DTS, Desktop Direct Transmission System, Scottsdale, Arizona, USA) with a sampling frequency of 4 kHz [33]. In parallel with EMG acquisition, the participant movements were monitored to assess changes in knee flexion angles over time. The knee flexion angle was determined indirectly by measuring the distance between the markers placed on the participant’s ankle and hip, followed by calculation using the law of cosines. Prior to data collection, each participant performed a 500-meter warm-up on the rowing ergometer, following established guidelines [34] to familiarise themselves with the rowing motion pattern. This was followed by a 10-minute rest period. Whereas the participants rowed on the ergometer until reaching their maximum subjective level of fatigue, subjective fatigue ratings were recorded every minute using the Borg Rating of Perceived Exertion (RPE) scale [30] while maintaining a stroke rate pace of 30 strokes per minute, controlled by an auditory cue. Each participant completed three test sessions. The overall experimental setup, including equipment configuration and signal processing, was kept consistent with methodologies employed in our previously related work [29,30] to ensure a valid comparison between the non-VR and VR conditions.

### VR scenery

**Fig 2:**
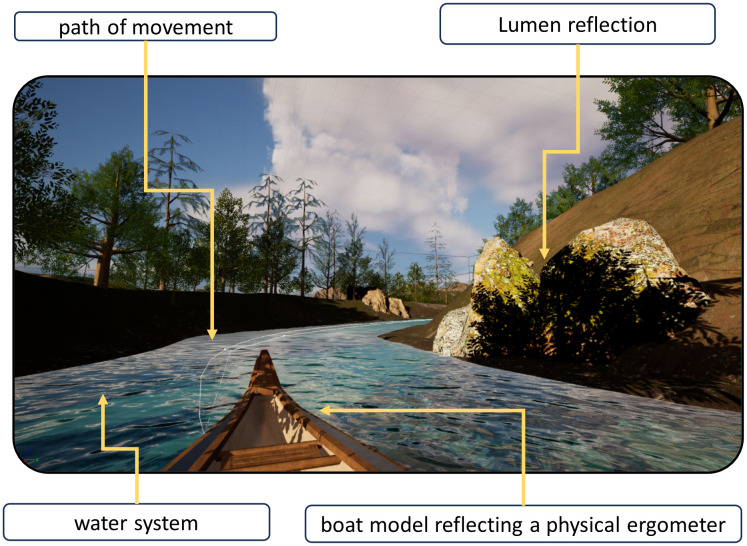
VR scene designed by the authors for conducting tests using the rowing ergometer.

The VR scene used for this study was developed using Unreal Engine 5 (UE), a game engine that enables the creation of highly realistic virtual environments. UE as a tool for creating scenarios has been referenced in numerous scientific articles across various research domains [35–37]. The software was selected for its ability to produce a realistic representation of the chosen scenery. UE 5 integrates advanced graphical technologies such as Nanite, which allows for detailed microgeometry rendering [38], and Lumen, a dynamic global illumination system [39]. They both significantly enhance the visual quality of virtual environments maintaining efficient computational performance. An example of the scene designed by the authors for conducting tests using the rowing ergometer is presented in Fig 2.

The scene constructed in Unreal Engine UE represents a river flowing through a forested landscape. The forest includes a range of 3D elements such as trees, bushes, grasses, rocks, and other ground vegetations. The 3D assets were sourced from the Quixel Megascans library, which offers a comprehensive collection of high-quality 3D models, textures, and materials [40]. The Water System module in UE 5 was used to simulate the river flow, contributing to a realistic depiction of water movement. To further enhance immersive quality of the environment, ambient soundtracks featuring natural background sounds were integrated.

During the design phase of the VR environment, particular attention was given to optimise the simulation to minimise the risk of cybersickness symptoms such as dizziness and nausea [41]. The application was compiled to maintain a refresh rate of at least 100 frames per second (fps), which required addressing key performance challenges associated with hardware limitations (Intel® Core™ i9-9900K CPU, 32 GB RAM, NVIDIA GeForce RTX 2070). The continuous rowing motion along the virtual river demanded dynamic rendering of scene objects synchronised with each stroke. To prevent frame rate drops, low-poly models from EpicGames’ free asset libraries were used, ensuring smooth visual performance during rowing exercises without noticeable interruptions caused by frame rate fluctuations.

The VR environment was also designed with user-controlled movement. A simplified kayak model was developed and integrated into the virtual river scene, enabling users to navigate the scene with control over speed and direction. The motion system was implemented using blueprint scripting, which made it possible to build a dynamic and adjustable speed control mechanism. This was achieved employing the spline function to define control points along the river path. The spline system utilises linear interpolation between these points, resulting in smooth and continuous motion that enhances both realism and interactivity within the VR environment [42].

### Calculations procedure

The calculations were based on data collected from two variants of the experiment: one conducted without the use of a VR (as reported in the work [29]) and the other incorporating a VR environment as part of the current study.

**Fig 3:**
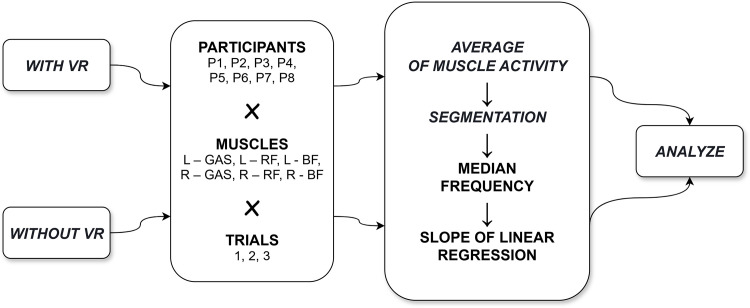
The modified algorithm based on work [29].

In both experimental variants, each participant performed three repetitions of the test. Based on the collected data, the calculations were carried out using the proposed method employing wavelet analysis. The algorithm applied in this process is presented in Fig 3.

The EMG signal processing algorithm was identical for both variants of the study. Each EMG signal was processed independently, following a four-step algorithm. A detailed description of each step is available in the publication [29], where the linear regression slope coefficients for the median frequency (MDF) were calculated in a non-VR study. In this study, the dataset was expanded to include EMG recordings obtained during VR experiments and these additional data were subjected to the same analytical procedure.

### Statistical power and effect size analysis

To supplement the statistical comparison, the effect size (Cohen’s d) and statistical power (1–β) were calculated using G*Power 3.1.9.7 software. The analysis was based on a two-tailed Wilcoxon signed-rank test for paired samples, consistent with the within-subject design (comparison of VR vs. non-VR conditions in the same participants, N = 8). Cohen’s d values were computed from the mean and standard deviation of paired differences between the two conditions using the formula:


$$d=\frac{M_{VR}-M_{non-VR}}{{SD}_{pool}}$$ (1)

where $M_{VR}\;and\;M_{non-VR}\;$ represent the mean MDF slope values for the VR and non-VR conditions, respectively, and *SD_pool_* is the pooled standard deviation derived from both conditions. Because the study used a within-subject design, the effect size correction for dependence was applied according to Morris and DeShon (2002) [43].

The calculated effect sizes were then entered into G*Power 3.1.9.7 to determine the achieved power at the significance level α = 0.05. The obtained power values allowed for the estimation of the probability of correctly detecting a true effect of the observed magnitude, thereby indicating the potential risk of a Type II error (β). To aid interpretation, Cohen’s d values were classified according to conventional benchmarks: d ≈ 0.2 (small), d ≈ 0.5 (medium), and d ≥ 0.8 (large). Values below 0.2 were considered negligible. These thresholds provide a qualitative assessment of the practical significance of the observed differences, even when statistical significance is not achieved.

## Results

**Fig 4:**
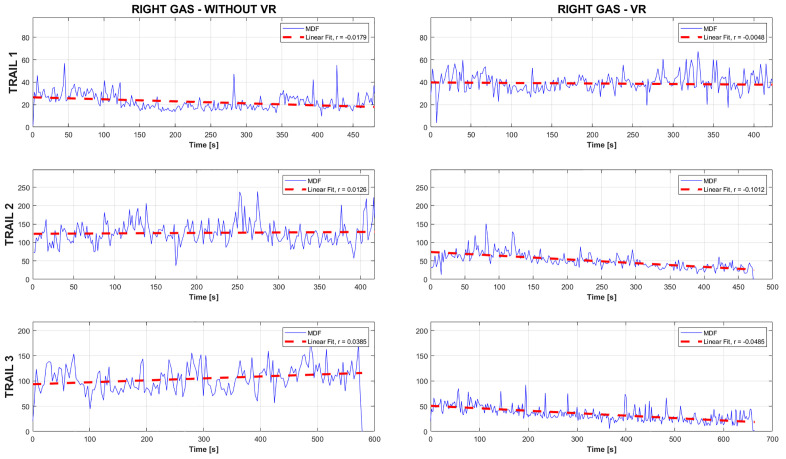
Sample MDF distribution for a selected muscle, comparing trials conducted with and without VR.

Following the methodology outlined in the preceding section, a series of calculations was performed to determine the slope coefficients of the linear regression for the median frequency MDF derived from the recorded EMG signals. For each participant, the results were divided into two distinct sets. The first set included data from trials conducted without the use of a VR environment, while the second set comprised data from trials in which VR was applied. This approach enabled a comparative analysis of muscle fatigue in different experimental conditions. To illustrate the obtained results Fig 4 presents a sample MDF distribution for one of the analysed muscle groups.

The graph presents sample MDF distributions together with their corresponding linear regression lines for one of the participants, based on EMG data recorded from the GAS muscle of the right lower limb. The left panel shows the results of three trials conducted under non-VR conditions, whereas the right panel illustrates the outcomes of three trials performed

in a simulated natural environment using VR. This approach enabled the calculation of linear regression coefficients for MDF across different muscle groups (GAS, BF, RF) for all the participants.

Subsequently, data analysis was carried out in two stages. The first ones involved the application of statistical tests comparing the results reported in the previous study [29] with those obtained in the current VR-based experiment, analysed for each participant individually. The second stage focused on collective data analysis, comparing the results of the entire group under conditions without VR with those with VR.

Statistical analysis was performed using the Wilcoxon signed-rank test to compare the fatigue indices (median MDF slope coefficients derived from the three trials) between the non-VR and VR conditions for each participant (N = 8). Separate tests were conducted for each of the six muscle measurement sites: Left RF, Right RF, Left BF, Right BF, Left GAS, and Right GAS. The significance level was established at α = 0.05.

The Wilcoxon test was conducted for each unit to identify individual responses to VR. The results of this test are presented in Table 1.

**Table 1:** The Wilcoxon signed-rank test for each unit.

	RF		BF		GAS	
	Left	Right	Left	Right	Left	Right
**Wilcoxon Statistic**	69	93	57	111	67	172
**p-value**	0.10985	0.44447	0.07618	0.88943	0.09524	0.0516

The results of the Wilcoxon signed-rank tests comparing non-VR and VR conditions for each muscle group indicate no significant differences for any of the muscle groups tested: Left RF (W = 69, p = 0.10985), Right RF (W = 93, p = 0.44447), Left BF (W = 57, p = 0.07618), Right BF (W = 111, p = 0.88943), Left GAS (W = 67, p = 0.09524), and Right GAS (W = 172, p = 0.0516). Although some trends might be observed (e.g., p-values approaching significance for Left BF and Right GAS), none reached the predetermined threshold for statistical significance.

Eight men (N = 8) participated in the study, performing exercises on a rowing ergometer under two experimental conditions: with virtual reality (VR) and without VR.

To complement the statistical comparison, effect sizes (Cohen’s d) were calculated for each muscle group, with values ranging from 0.15 to 0.35, indicating small or negligible effects according to conventional benchmarks. Corresponding post hoc statistical power (1–β), estimated using G*Power 3.1 software, ranged from 0.38 to 0.45, suggesting a low probability of detecting a true effect and a high risk of Type II error (β).

These results indicate that the lack of statistical significance may reflect insufficient test power rather than the true absence of an effect, highlighting the need for caution when interpreting the findings and for larger sample sizes in future studies.

**Fig 5:**
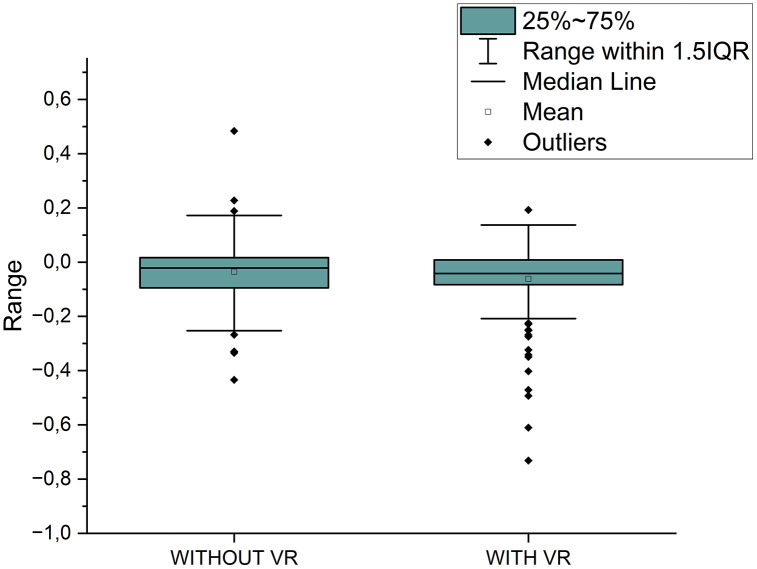
Variability range in muscle fatigue levels without VR and with VR within the group.

Fig 5 provides a comparison of muscle fatigue levels (for the muscle groups GAS, BF, and RF) in subjects with and without the use of VR technology. The plot illustrates the range of variability in muscle fatigue values between the two groups.

Fig 5 presents a comparison of muscle fatigue variability under two conditions: without and with VR using a boxplot. The range of values determined by 1.5 IQR appears very similar in both cases, indicating a comparable spread of data under non-VR and VR conditions. Both the mean and median values in the non-VR condition are slightly higher than those observed in the VR condition.

While the plot provides a visual overview suggesting a minimal overall difference and similar data spread (IQR), it is important to note that the formal statistical tests performed separately for each muscle group (Table 1) did not confirm any significant differences between the conditions.

### Study limitations

This study has several limitations that should be considered when interpreting the results. First, the small sample size (N = 8) limits the statistical power of the analysis and the generalizability of the findings. Although the participants were highly homogeneous, comprising trained male athletes from the same university rowing section, this homogeneity restricts the ability to extrapolate the results to broader or more diverse populations, such as females, untrained individuals, or athletes from different disciplines. Future studies should therefore include a larger and more heterogeneous group of participants to enhance the external validity of the results. This specific, highly-trained cohort, while ideal for reducing inter-subject variability, represents a unique athletic population, making direct comparisons to other challenging groups.

Second, the time interval of approximately six months between the non-VR and VR experimental phases, although controlled through continuous monitoring and standardised training, may have introduced uncontrolled variables such as subtle changes in muscle conditioning, psychological state, or motivation. These temporal factors could have influenced the EMG signal characteristics and fatigue patterns despite the precautions taken to maintain comparable physical performance levels.

Third, the study relied primarily on EMG-derived indices (MDF slope coefficients) to quantify muscle fatigue. While these parameters provide valuable objective measures, they do not fully capture the multidimensional nature of fatigue, which includes psychological, metabolic, and cardiovascular components.

Fourth, technical aspects of EMG measurement during dynamic exercise, such as electrode displacement, skin perspiration, or movement artifacts may have introduced variability in the recorded signals, potentially affecting the accuracy of the fatigue estimation. Although standardised procedures (e.g., SENIAM skin preparation and band-pass filtering) were applied, such methodological limitations are inherent to EMG studies involving movement.

Fifth, the study employed a single VR scenario depicting a natural river environment. While this provided a controlled and realistic simulation, it does not allow conclusions about how different types of VR environments (e.g., urban, competitive, or abstract) may differentially influence fatigue and motivation. Future studies should compare multiple virtual contexts to assess how environmental design features impact physiological and psychological responses.

Finally, despite efforts to minimise cybersickness through optimisation of frame rate and scene design, subjective responses to VR exposure were not formally evaluated. Including self-reported measures of immersion, presence or discomfort could have provided complementary insights into user’s experience and its potential impact on muscle fatigue outcomes.

In summary, while the study provides valuable preliminary insights into the effects of immersive VR on muscle fatigue during rowing, the aforementioned limitations underscore the need for future research with larger samples, broader environmental conditions and multimodal measurement approaches to more comprehensively understand the interaction between virtual immersion and neuromuscular performance.

## Discussion

Muscle fatigue is a physiological phenomenon characterised by a temporary reduction in muscle power during intense and repetitive myocyte contractions. Numerous studies have described methods for processing EMG signals to determine fatigue parameters, including Fast Fourier Transform (FFT), Continuous Wavelet Transform (CWT) and Discrete Wavelet Transform (DWT). Among these, the wavelet transform has been recognised as superior for analysing non-stationary signals compared to FFT, while DWT offers the additional advantage of reducing redundancy effects. The DWT-based method was therefore adopted in this study as the foundation for calculating the impact of VR on muscle fatigue.

Recent research examining fatigue in the context of VR [44] has primarily focused on psychological aspects, where participants report their sensations, motivation changes and perceived fatigue following exposure to virtual environments. These studies often include assessments of cybersickness symptoms as well. However, scientific investigations utilising VR for biofeedback have rarely incorporated quantitative measures of muscle fatigue derived from EMG data [27]. Moreover, in studies where muscle fatigue was analysed during dynamic movement using VR, DWT method was not applied for data analysis [29,30]. Although some research has explored how different environmental types influence muscle activity [28], direct quantitative comparison of the muscle fatigue with and without VR, using the methodology employed in the present work – remain scarce. This highlights the need for more studies specifically focused on quantitative assessment of muscle fatigue in VR environments [31].

The authors of this study aimed to examine whether, based on the results of the previously described experiment [29], which determined the fatigue parameters of the specified muscle groups (GAS, BF, RF) in eight participants, each tested in three repetitions, applying the same analytical algorithm [30], while introducing a VR component and maintaining identical measurement conditions with the same group of participants would yield different outcomes in terms of muscle fatigue. The objective was to assess whether VR influences fatigue levels in lower limb muscles. In this study a VR simulation depicting a natural environment was used with the intension of potentially reducing muscle fatigue by eliciting a relaxation effect through visual elements such as trees, water, and mountains. The hypothesis of a positive influence of the natural settings was derived from previous studies [17], which demonstrated participants’ preference for natural over urban scenarios. Considering that participants typically trained on a rowing ergometer indoors, their baseline perception of fatigue could have been influenced prior to the introduction of the VR conditions. Both experimental setups were designed to measure fatigue parameters of the same muscle groups (GAS, BF, RF) in the same eight participants, with each trial repeated three times. Following the application of the Wilcoxon signed rank test comparing the VR and non-VR conditions for each muscle group separately, no statistically significant differences were observed in any of the measured lower limb muscles (Table 1). These findings within the parameters of this experimental design, analytical approach and the immersive VR environment did not induce a statistically significant systematic change in the rate of muscle fatigue as assessed by sEMG MDF slope compared to the non-VR condition.

The Wilcoxon signed-rank test did not reveal statistically significant differences between the VR and non-VR conditions for any muscle group (p > 0.05). However, additional analysis of effect sizes (Cohen’s d) indicated small or negligible effects (d = 0.15–0.35). Corresponding post hoc statistical power (1–β = 0.38–0.45) was low, suggesting a limited probability of detecting true effects and a substantial risk of Type II error (β).

These results imply that the absence of statistical significance should not be interpreted as evidence of no effect. Rather, the combination of small effect sizes and low power indicates that subtle effects of VR on muscle fatigue may exist but could not be reliably detected in the current sample of eight participants.

The lack of significant findings may also suggest that for highly trained athletes involved in physically demanding disciplines such as rowing, intrinsic factors such as strong self-awareness of effort regulation and well-developed pacing strategies exert a greater influence on physiological outcomes than the surrounding visual scenery. This effect might be even more pronounced in controlled laboratory conditions where external motivators, such as direct competition between participants or ergogenic aids were absent, as was the case in both the present VR study and the referenced non-VR experiment. Certain study-specific factors should also be considered. Variables such as participants’ individual characteristics of their prior familiarity with VR technology and their overall health or well-being status during the study period could have significantly influenced the results. Although minor differences were observed in the median, mean, and spread of muscle fatigue values the quantitative EMG-based analysis did not reveal statistically significant variations between the VR and non-VR conditions. This indicates that any potential psychologically induced by the VR environment did not translate into measurable and statistically significant alteration of the EMG-derived fatigue index for the lower limb muscles during this rowing task.

### Future work

These results are intriguing; however, they should be interpreted with caution due to potential confounding factors. The dynamic nature of exercise introduces specific methodological challenges, such as skin displacement and perspiration, which may affect the accuracy and reliability of EMG recordings. To mitigate the impact of these factors all the experimental sessions were conducted in a climate-controlled laboratory with the ambient temperature. A comprehensive understanding of VR’s potential impact on muscle fatigue requires future research involving larger participant groups and a broader range of controlled variables. Although the sample size in this study was limited, it encompassed the entire sports section of the facility and was homogenised according to previous criteria. Nevertheless, future studies including larger and more diverse samples would enhance both the credibility and generalizability of the findings. Future work should also aim to investigate factors more systematically, including the influence of external motivators such as direct competition and how individual characteristics, such as familiarity with VR, or providing the real-world training procedures (what has an impact on heat and sweating) may modulate the relationship between virtual environments and physiological outcomes. It is also worth considering an alternative method to the subjective fatigue assessment in future work. Integrated physiological measurement systems capable of monitoring VO_2_ and HR [45] could provide complementary physiological data to better characterise maximal fatigue. While subjective ratings of perceived exertion remain widely used, they may reduce objectivity and precision when determining the point of maximal fatigue. Moreover, establishing standardised methodologies for assessing muscle fatigue in dynamic exercises using EMG would significantly strengthen analytical robustness and facilitate broader comparative studies. Such standardisation would allow researchers to apply consistent approaches across different populations and sports disciplines. The participants in this study did not report adverse effects related to VR exposure, and experiment’s duration was consistent with previous research [46], remaining below the threshold typically associated with negative VR-related symptoms (i.e., 20 minutes). From a methodological perspective, future investigations should aim to reduce the temporal gap between data collection for comparative experimental conditions when employing within-subject design. The use of randomised crossover or counterbalanced sequence for the experimental conditions (e.g., VR vs. non-VR) is also recommended to minimise potential order effects. Furthermore, integrating multi-modal data streams, such as physiological signals (e.g., sEMG), kinematic parameters and interaction metrics within immersive virtual environments could provide a valuable foundation for the future application of artificial intelligence methods. These approaches may facilitate the development of predictive models of neuromuscular fatigue and support the personalisation of training protocols. Finally, collecting participant feedback during experimental sessions would offer complementary insights. A potential direction for expanding future research involves the use of tools such as fNIRS (Functional Near-Infrared Spectroscopy) to monitor both muscle and cerebral oxygenation. However, when implementing such multimodal systems, it will be necessary to address the technical challenges associated with integrating fNIRS with other measurement devices including VR and EMG systems.

## Conclusions

The primary research question of this study was to determine if an immersive, naturalistic virtual reality (VR) environment would significantly alter the progression of neuromuscular fatigue in the lower limb muscles (GAS, BF, RF) during a dynamic rowing task, compared to an identical task performed without VR. Fatigue was quantitatively assessed using sEMG signal analysis based on the Discrete Wavelet Transform (DWT) and the slope of the Median Frequency (MDF).

Based on the quantitative analysis of sEMG signals using DWT and MDF slope calculation and using a statistical approach testing each muscle group separately, this study did not find statistically significant differences in muscle fatigue progression between rowing exercise performed with and without immersive naturalistic VR environment (p > 0.05). Therefore, the hypothesis that this specific VR environment would significantly affect muscle fatigue compared to a non-VR condition was not supported by the EMG data analysis.

While the primary finding was a lack of statistical significance, this result has practical implications. For sport performance, it suggests that for highly-trained athletes, a passive, naturalistic VR environment may not be a sufficient stimulus to alter underlying physiological fatigue patterns. For rehabilitation, the absence of a significant difference in fatigue implies that VR can be safely implemented as a motivational and engagement tool to increase patient adherence to repetitive exercises without necessarily inducing greater physiological strain. For VR development, this study highlights that future systems aiming to actively modulate fatigue should move beyond passive scenery and integrate more interactive elements, competition, or real-time biofeedback, paving the way for the AI-driven personalised training paradigms mentioned in our future work.

However, it is important to acknowledge that the absence of significance does not necessarily preclude the existence of a real-world effect of VR on muscle fatigue. Furthermore, an examination of the specific results (Table 1) reveals that a majority of the calculated p-values were relatively close to the conventional significance threshold (often p ≤ 0.11). This proximity suggests potential underlying trends that narrowly missed statistical confirmation and underscore the need for further investigation.

Furthermore, the combination of VR with EMG systems requires further research in this area [31]. The integration of VR with EMG presents a promising method for analysing muscle fatigue, particularly when fatigue is quantified using appropriately selected analytical methods, which remains a debated issue in muscle fatigue research employing EMG [29,30]. Further research with potentially larger sample sizes, different populations, or alternative metrics is important to fully clarify the relationship between VR immersion and neuromuscular fatigue during dynamic exercise. In addition, such studies may find applications in various sports, potentially providing future insight that enables the comparison of fatigue levels depending on different parameters.

In conclusion, while this study did not detect statistically significant effects of VR on lower limb muscle fatigue, the inclusion of Cohen’s d and post hoc power analysis provides valuable context, indicating that small effects may exist but were not detectable due to limited sample size. These findings underscore the importance of effect size and power considerations in VR and EMG research, particularly when studying highly trained athletes with low variability in physiological responses.

## Supporting information

S1 File Data file 1. (CSV)

S2 File Data file 2. (CSV)
